# 572. Adverse Events Following Live Immunization in Patients with DiGeorge Syndrome: A Retrospective, Single Center Study in Korea

**DOI:** 10.1093/ofid/ofac492.625

**Published:** 2022-12-15

**Authors:** Sung Min Lim, Je Hee Shin, Jee Yeon Baek, Ji Young Lee, Ji-Man Kang, Jong Gyun Ahn

**Affiliations:** Severance Children’s Hospital, Yonsei University College of Medicine, Seoul, Seoul-t'ukpyolsi, Republic of Korea; Severance Children’s Hospital, Yonsei University College of Medicine, Seoul, Seoul-t'ukpyolsi, Republic of Korea; Yonsei University College of Medicine, Seoul, Seoul-t'ukpyolsi, Republic of Korea; Yonsei University College of Medicine, Seoul, Seoul-t'ukpyolsi, Republic of Korea; Severance Children’s Hospital, Yonsei University College of Medicine, Seoul, Seoul-t'ukpyolsi, Republic of Korea; Severance Children’s Hospital, Yonsei University College of Medicine, Seoul, Seoul-t'ukpyolsi, Republic of Korea

## Abstract

**Background:**

DiGeorge syndrome (DGS) is a syndrome accompanied by congenital heart defect, hypoparathyroidism and immunodeficiency of varying severity. Live vaccination is generally contraindicated in patients with DGS. However, in real clinical practice, there are cases in which live vaccines are immunized before the diagnosis of DGS or are incidentally immunized. We collected these cases and investigated adverse events (AEs), especially infections caused by the vaccine strains.

**Methods:**

This retrospective study included all patients diagnosed with DGS at Severance Hospital Seoul, Korea, between November 2005 and June 2021. We extracted patients with ICD-10 code (D82.1) and then excluded subjects without genetic confirm. According to the immune status, subjects were categorized into three groups: group A [CD3 < 500 or CD8 < 200 (cells/mm3)], group B [CD3 ≥500 and CD8 ≥ 200 (cells/mm3)] and group C (unknown).

**Results:**

Of a total 94 DGS patients, approximately 40% of subjects (38/94) underwent immunological test, of which 21% (8/38) belonged to group A and 79% (30/38) were in group B. Approximately 80% of study subjects (73/94) had a record of at least one live vaccination. By vaccine type, measles-mumps-rubella accounted for the most at 70% (66/94), followed by varicella, bacillus Calmette–Guérin, rotavirus, and live-attenuated Japanese encephalitis virus vaccine. A total of 50 AEs were observed (Figure 1): fever (n=29), URI (n=9), diarrhea (n=4), rash (n=3), thrombocytopenia (n=3), injection site pus (n=1) and febrile convulsion (n=1). Among them 26% (13/50) occurred in group A and no significant difference in the incidence rate of AEs between group A and B was observed (*P* = 0.14). Six cases of them turned out to be consistent with live vaccination by WHO 2009 causality analysis. Moreover, there were no serious reactions, including ICU hospitalization or death, and there was no emergence of disease caused by vaccine strains.

**Figure:**
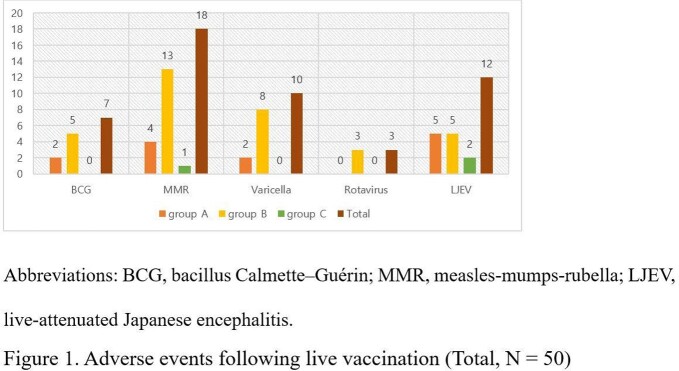

**Conclusion:**

Our data show that live vaccines were often given without immunologic screening and generally well tolerated in our study population.

**Disclosures:**

**All Authors**: No reported disclosures.

